# Supplementary material for: Protein analysis of extracellular vesicles to monitor and predict therapeutic response in metastatic breast cancer
Source: Nat Commun. 2021 May 5;12:2536. doi: 10.1038/s41467-021-22913-7 (PMC8100127; doi:10.1038/s41467-021-22913-7)
Supplement: Supplementary file 1 — Supplementary Information [file 41467_2021_22913_MOESM1_ESM.pdf]

SUPPLEMENTARY INFORMATION

Protein analysis of extracellular vesicles to monitor and predict therapeutic response in metastatic breast cancer

Tian et al.

Supplementary Information Contains:

Supplementary Figures 1-17

Supplementary Tables 1-16

Supplementary Data

Supplementary Software

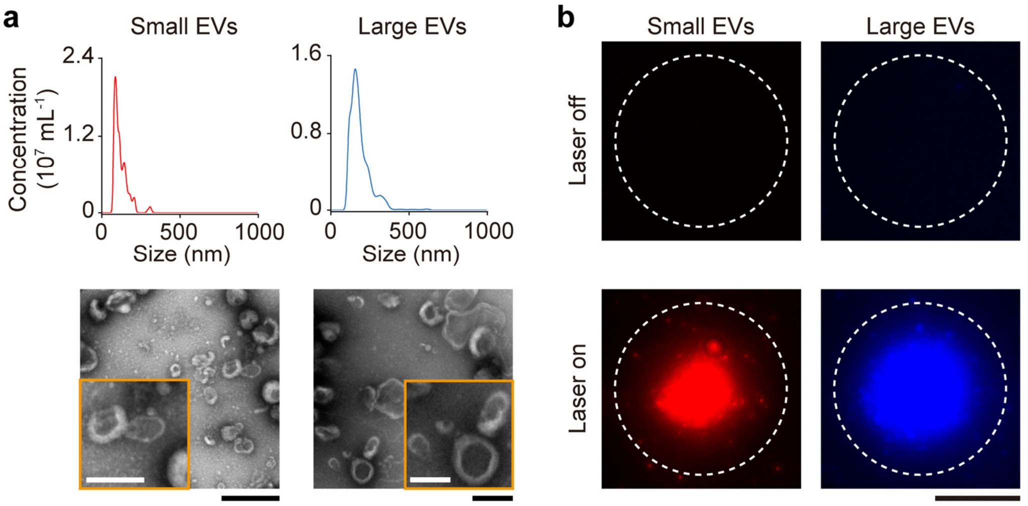

Supplementary Fig. 1 | Thermophoretic accumulation of small EVs and large EVs. a, TEM and NTA characterization of small EVs (red line) and large EVs (blue line). Representative images are shown from three independent repeats. Scale bars, 500 nm (insert, 250 nm). **b,** Fluorescence images of DiO-labelled small EVs (10^{10} mL^{-1}) and large EVs (8×10^{9} mL^{-1}) after 10 min laser irradiation. Representative images are shown from two independent repeats. Scale bar, 50 µm. Source data are provided as a Source Data file.

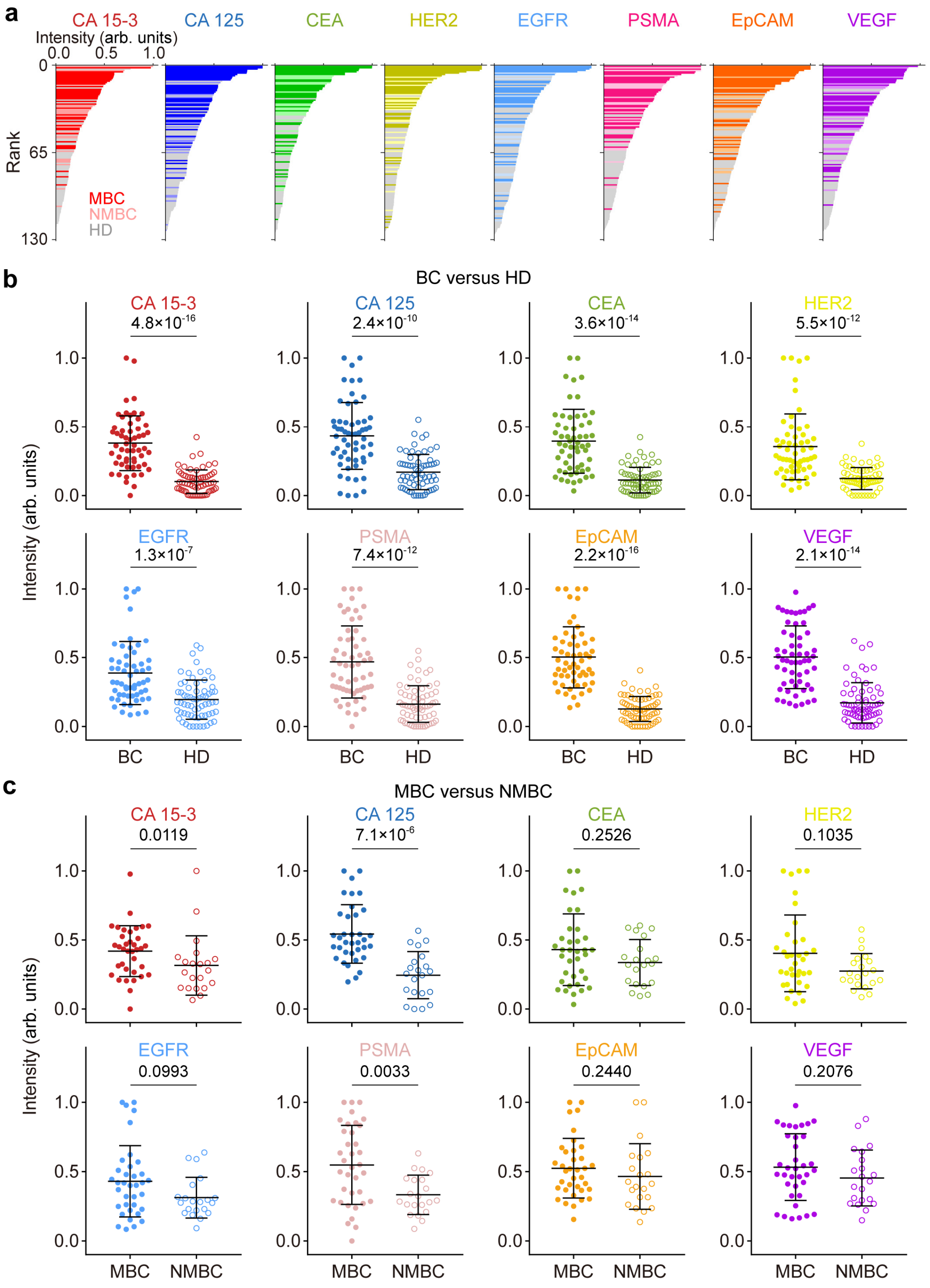
a
CA 15-3
CA 125
CEA
HER2
EGFR
PSMA
EpCAM
VEGF
Intensity (arb. units)
0.0 0.5 1.0
Rank
0
65
130
MBC
NMBC
HD
b
BC versus HD
CA 15-3
4.8×10-16
CA 125
2.4×10-10
CEA
3.6×10-14
HER2
5.5×10-12
EGFR
1.3×10-7
PSMA
7.4×10-12
EpCAM
2.2×10-16
VEGF
2.1×10-14
Intensity (arb. units)
1.0
0.5
0.0
BC HD
c
MBC versus NMBC
CA 15-3
0.0119
CA 125
7.1×10-6
CEA
0.2526
HER2
0.1035
EGFR
0.0993
PSMA
0.0033
EpCAM
0.2440
VEGF
0.2076
MBC NMBC

Supplementary Fig. 2 | Performance of individual EV markers in BC versus HD discrimination and MBC versus NMBC discrimination. a, Waterfall plots showing the expression levels of 8 EV protein markers sorted from high (top) to low (bottom). **b,** Elevated expression levels of all 8 EV protein markers in plasma samples from BC patients ($n = 57$, solid dots) compared with HD ($n = 66$, void dots). **c,** Expression levels of 8 EV protein markers in plasma samples from MBC patients ($n = 36$, solid dots) and NMBC patients ($n = 21$, void dots). Statistical differences were determined by two-sided, nonparametric Mann-Whitney test (**b**,**c**). P values are indicated in the charts. Error bars represent the mean ± s.d. in (**b**, **c**). Source data are provided as a Source Data file.

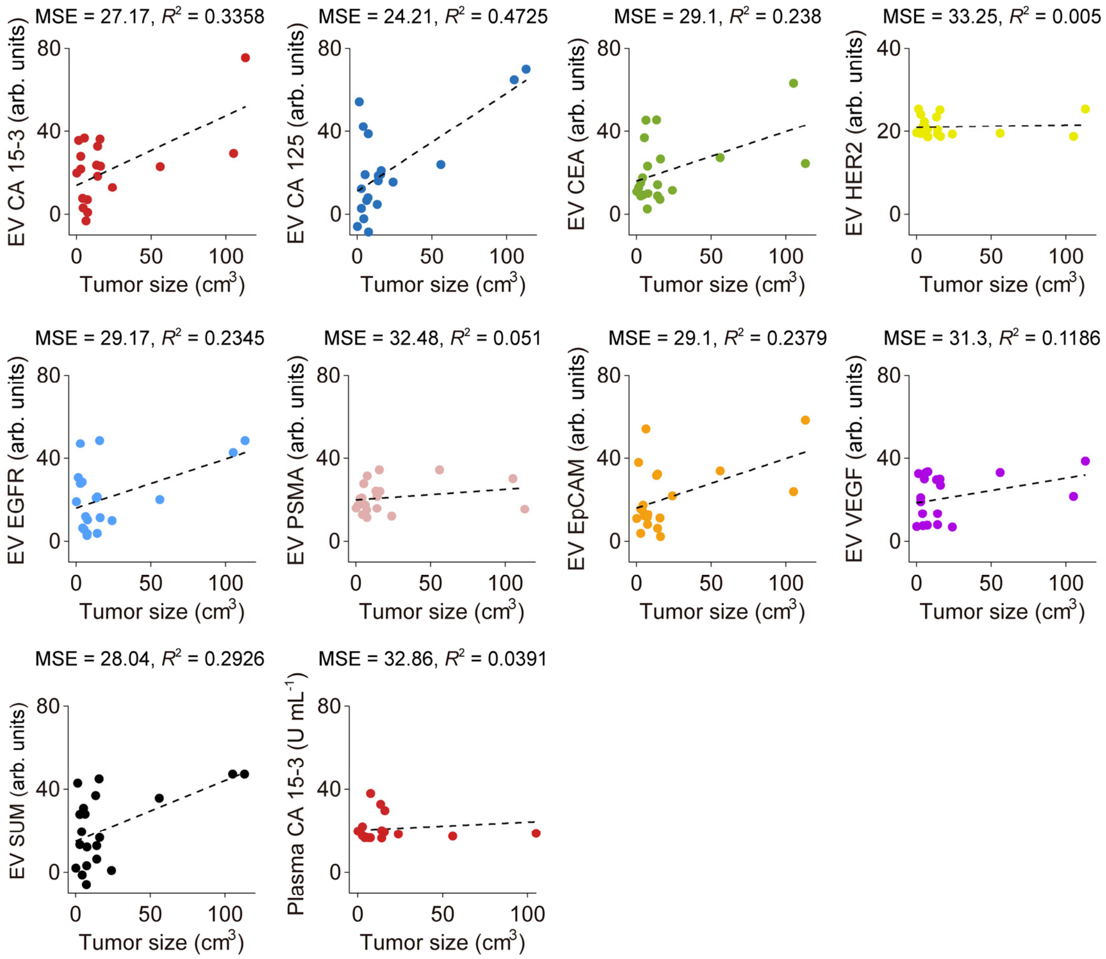

Supplementary Fig. 3 | Correlation between tumor size and EV or plasma protein markers using linear regression. Tumor sizes measured in pretreatment MBC patients (n = 20) versus the expression of 8 individual EV markers, SUM, and plasma CA 15-3. Mean square errors (MSE) and R squared (R^2) are indicated. Linear regression result is indicated by the dashed line. Source data are provided as a Source Data file.

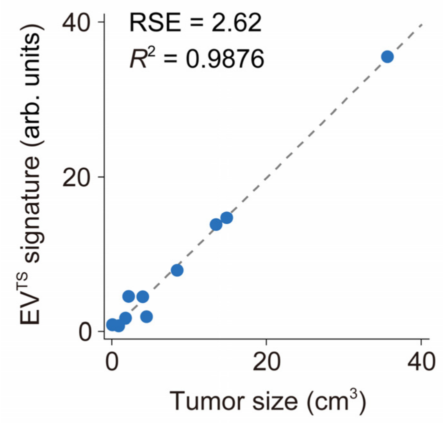

Supplementary Fig. 4 | Concordance between primary tumor size (*n* = 11) and the EV^{TS} signature identified using multivariate linear regression. Mean square errors (MSE) and R squared (R^2) are indicated. Linear regression result is indicated by the dashed line. Source data are provided as a Source Data file.

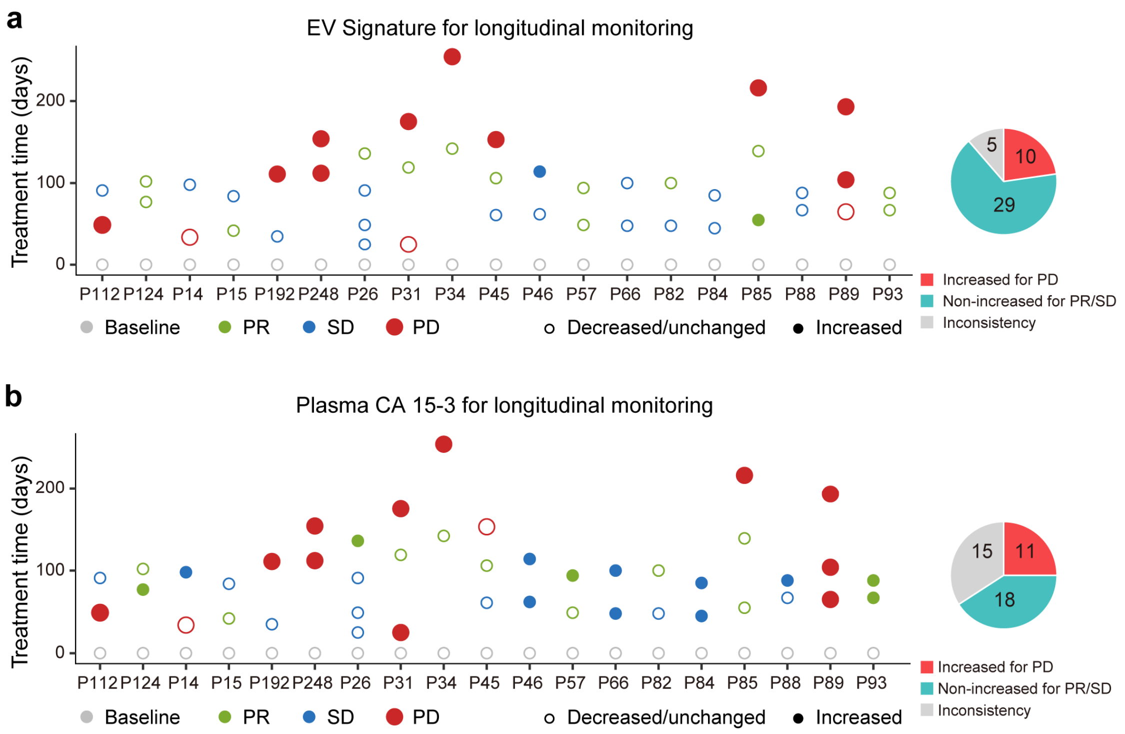

Supplementary Fig. 5 | Performance of the EV^{M} signature and plasma CA 15-3 for longitudinal monitoring of therapeutic responses of MBC patients. a, The EV^{M} signature showing consistency with therapeutic responses (increased for PD or decreased/unchanged for PR/SD) in 88.6 % of cases (39/44) from 19 MBC patients. **b,** The relative change in plasma CA 15-3 level between two time points showing consistency with therapeutic responses in 65.9 % of cases (29/44) from 19 MBC patients. The solid green, blue, and red dots indicate that the value of EV^{M} signature was increased at PR, SD, and PD, respectively. The void gray, green, blue, and red dots indicate that the value of EV^{M} signature was decreased or unchanged at the baseline, PR, SD, and PD, respectively. Source data are provided as a Source Data file.

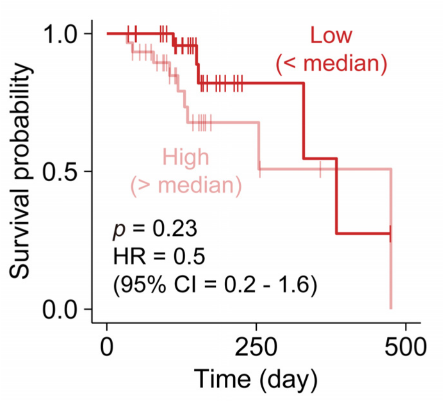

Supplementary Fig. 6 | Plasma CA 15-3 for prediction of progression-free survival (PFS) in a MBC cohort. Kaplan-Meier curves showing PFS of 59 MBC patients according to the plasma CA 15-3 level before treatment (baseline). The baseline level (high or low) was stratified according to the median value. The significance of difference was calculated by two-sided log-rank test. Hazard ratio (HR) and 95% CI were calculated using Cox proportional-hazard regression with a univariate model. Source data are provided as a Source Data file.

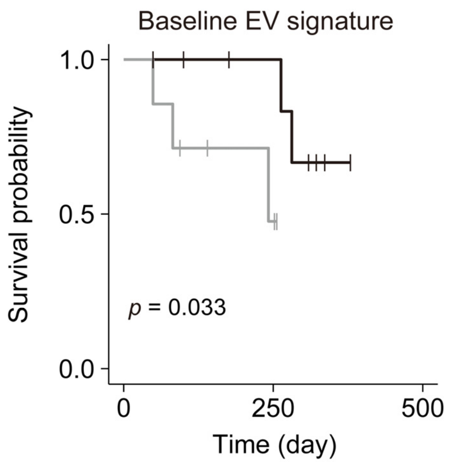

Supplementary Fig. 7 | EV^{P} signature for prediction of progression-free survival (PFS) in a prospective cohort. Kaplan-Meier curves showing PFS of 15 MBC patients according to the EV^{P} signature before treatment (baseline). The baseline level (high or low) was stratified according to the median value. The significance of difference was calculated by two-sided log-rank test. Source data are provided as a Source Data file.

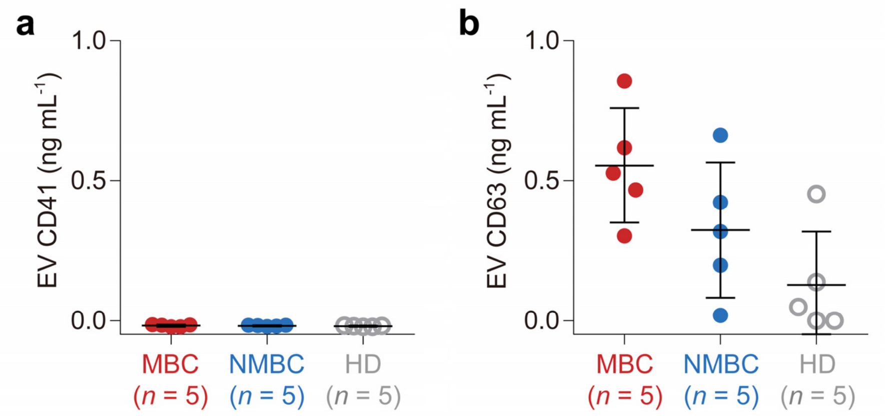

Supplementary Fig. 8 | ELISA measurement of the expression level of EV CD41 and EV CD63 for MBC, NMBC, and HD. EVs were isolated from plasma samples of MBC patients (n = 5, red dots), NMBC patients (n = 5, blue dots) and HDs (n = 5, gray dots) by ultracentrifugation. Error bars represent the mean ± s.d. in (**a**, **b**). Source data are provided as a Source Data file.

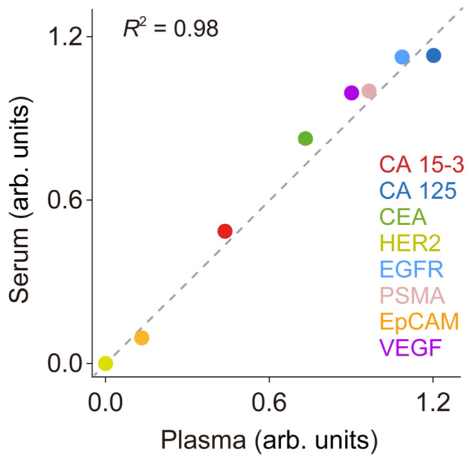

Supplementary Fig. 9 | TAS measurement of EV spiking samples. MDA-MB-231 EVs (10^{10} mL^{-1}) were spiked in EV-depleted serum and EV-depleted plasma samples from the same individual, and the expression levels of 8 EV surface markers in serum and plasma were measured by TAS. R square (R^2) is indicated. Source data are provided as a Source Data file.

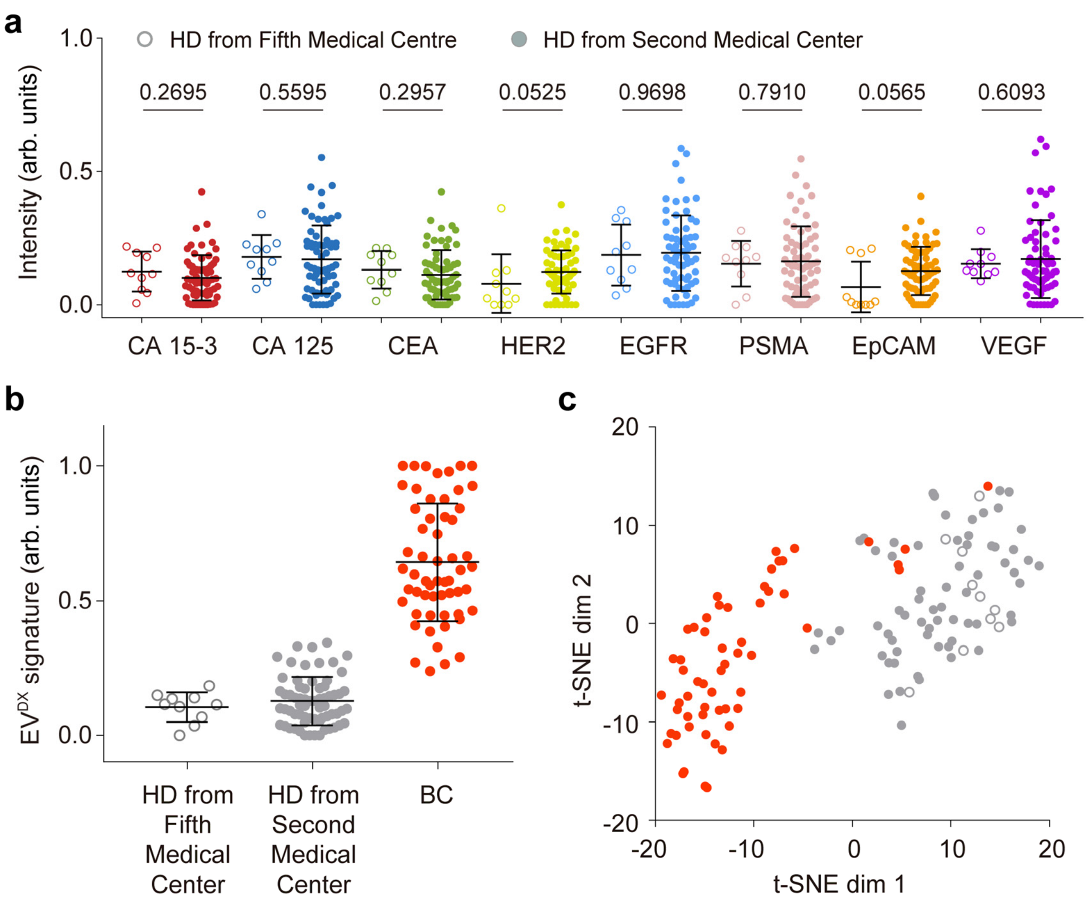

Supplementary Fig. 10 | TAS detection of EV surface proteins in plasma samples from two different hospitals. a, Expression levels of 8 EV surface proteins in plasma samples collected from Fifth Medical Center (n = 10, void dots) and Second Medical Center (n = 66, solid dots). Statistical differences were determined by two-sided Mann-Whitney test. **b-c,** Values of EV^{DX} signature (**b**) and t-SNE plot (**c**) showing that all the HDs from Fifth Medical Center can be correctly differentiated from BC patients. Void gray dots represent HD from Fifth Medical Center (n = 10 individuals), solid gray dots represent HD from Second Medical Center (n = 66 individuals), and solid red dots represent BC patients from Fifth Medical Center (n = 57 individuals) in (**b**, **c**). Error bars represent the mean ± s.d. in (**a**, **b**). Source data are provided as a Source Data file.

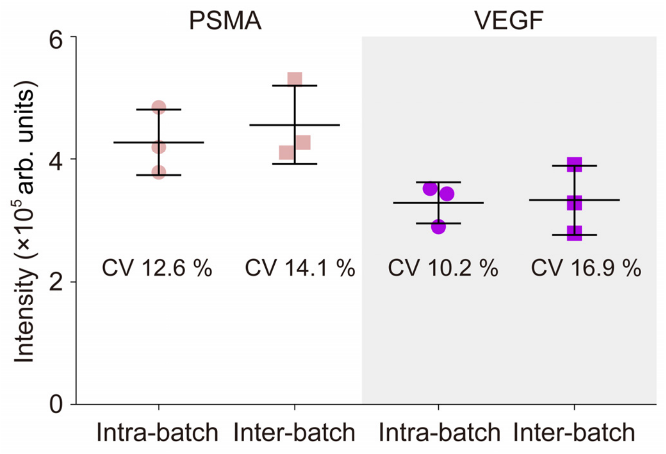

Supplementary Fig. 11 | Repeatability and Reproducibility of TAS for detecting plasma EVs. Intra-batch variation (circles) of TAS assay was determined by measuring the expression of EV PSMA (pink) and VEGF (violet) from the same plasma sample 3 times in 1 run. Inter-batch variation (squares) was determined by detecting plasma EVs (n = 3) at different time points. Inter- and intra-batch variations were defined as the ratio of the standard deviation to the mean value. CV, coefficient of variation. Error bars represent the mean ± s.d.. Source data are provided as a Source Data file.

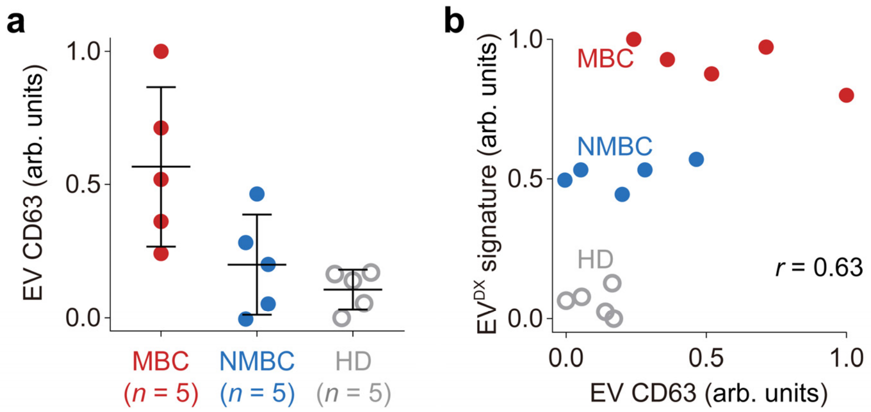

Supplementary Fig. 12 | Analysis of EV CD63 by TAS. a, Expression levels of CD63 on plasma EVs from MBC patients (n = 5, red dots), NMBC patients (n = 5, blue dots), and HD (n = 5, gray dots). **b,** Correlation analysis between EV CD63 and the EV^{DX} signature. Error bars represent the mean ± s.d. in (**a**). Pearson correlation coefficient r is indicated in (**b**). Source data are provided as a Source Data file.

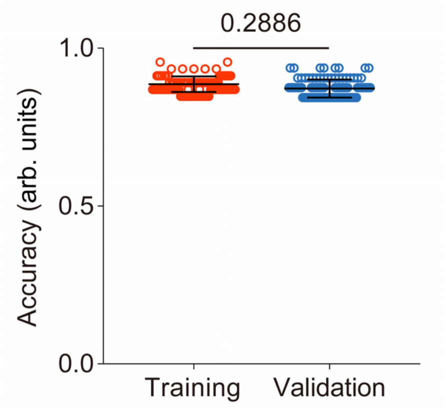

Supplementary Fig. 13 | Accuracy for PD versus PR/SD classification in 100 sets training and validation cohorts. Statistical differences were determined by two-sided, paired *t* test. Red dots represent accuracies for the training cohort (*n* = 100 sets) and blue dots for the validation cohort (*n* = 100 sets). Error bars represent the mean ± s.d.. Source data are provided as a Source Data file.

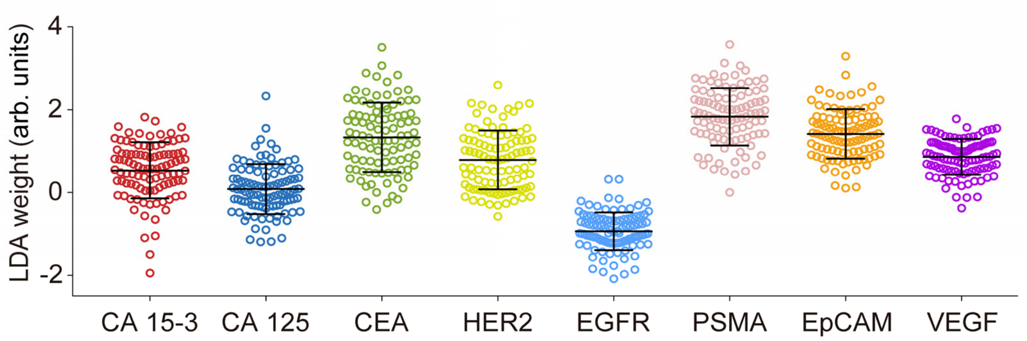

Supplementary Fig. 14 | Variable contribution of different EV markers to the EV^{M} signature. LDA weights of 8 EV markers from 100 iterations of training sets (n = 100 sets for each EV marker). Error bars represent the mean ± s.d.. Source data are provided as a Source Data file.

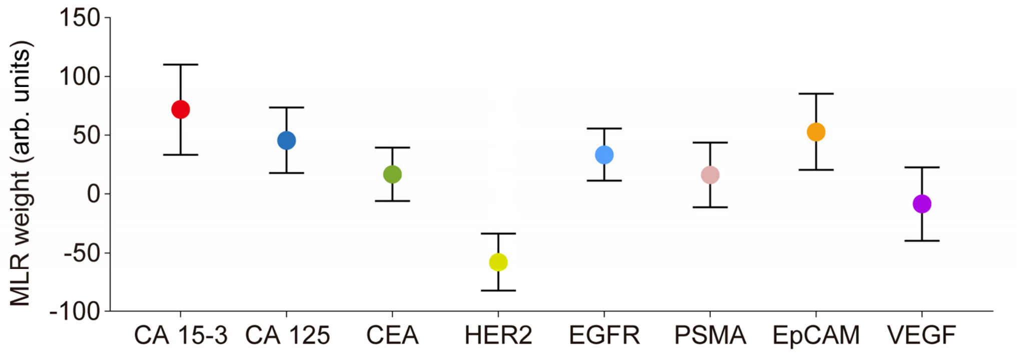

Supplementary Fig. 15 | Variable contribution of different EV markers to the EV^{TS} signature. n = 1 set of MLR for each EV marker. Points represent the MLR weights and error bars represent the 95% CI. Source data are provided as a Source Data file.

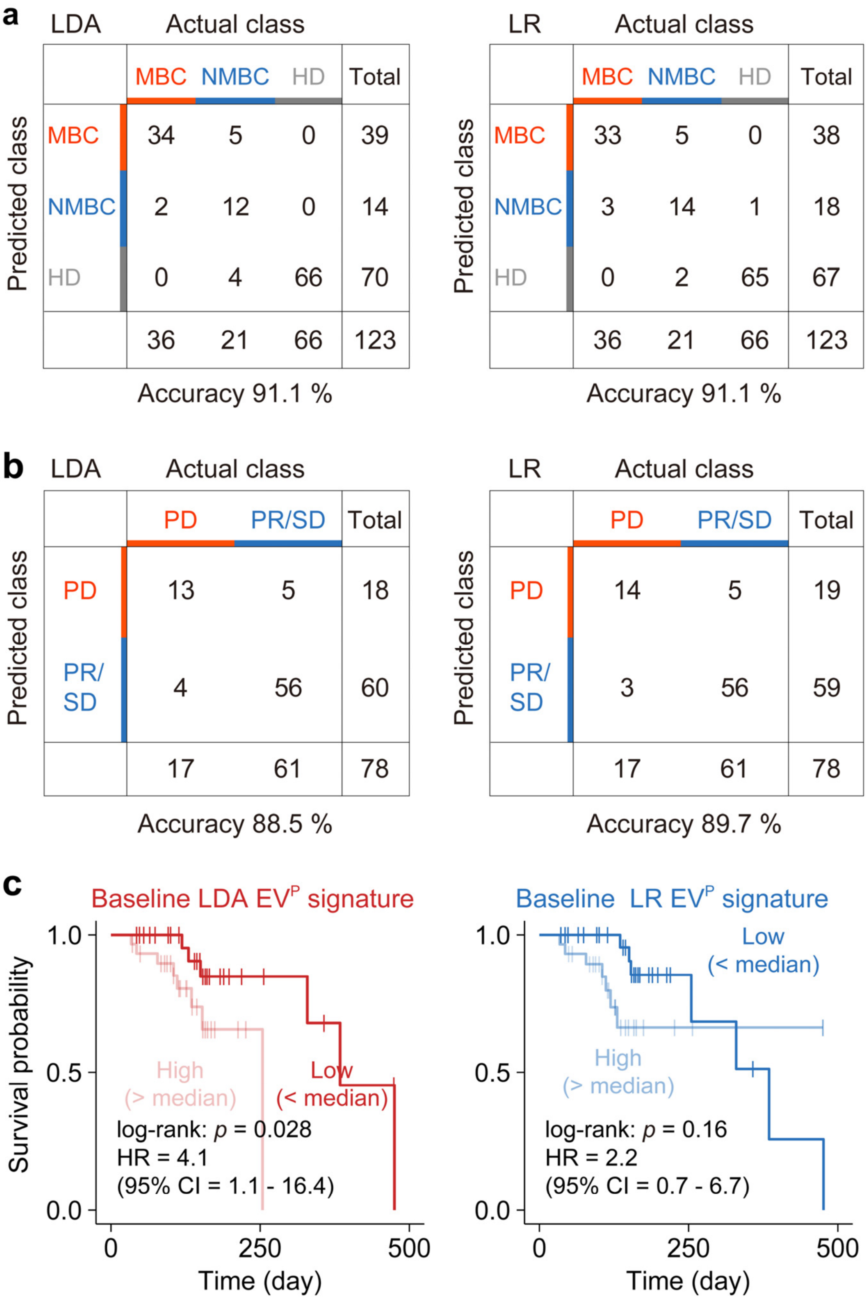

Supplementary Fig. 16 | Comparison of LDA and logistic regression (LR) classifiers in MBC diagnosis, treatment response monitoring, and prognostic prediction. a, Confusion matrix showing an overall accuracy of 91.1 % across MBC (n = 33), NMBC, (n = 21), and HD (n = 66) using both LDA and LR classifiers. **b**, Confusion matrix showing that LDA and LR had similar accuracies of 88.5 % and 89.7 % in differentiating

PD (*n* = 17) from PR/SD (*n* = 61). **c**, Prediction of progression-free survival (PFS) in a MBC cohort by LDA and LR. Kaplan-Meier curves showing PFS of 59 MBC patients stratified according to the median value of the EV^P signature. The significance of difference was calculated by two-sided log-rank test. Hazard ratio (HR) and 95% CI were calculated using Cox proportional-hazard regression with a univariate model. Source data are provided as a Source Data file.

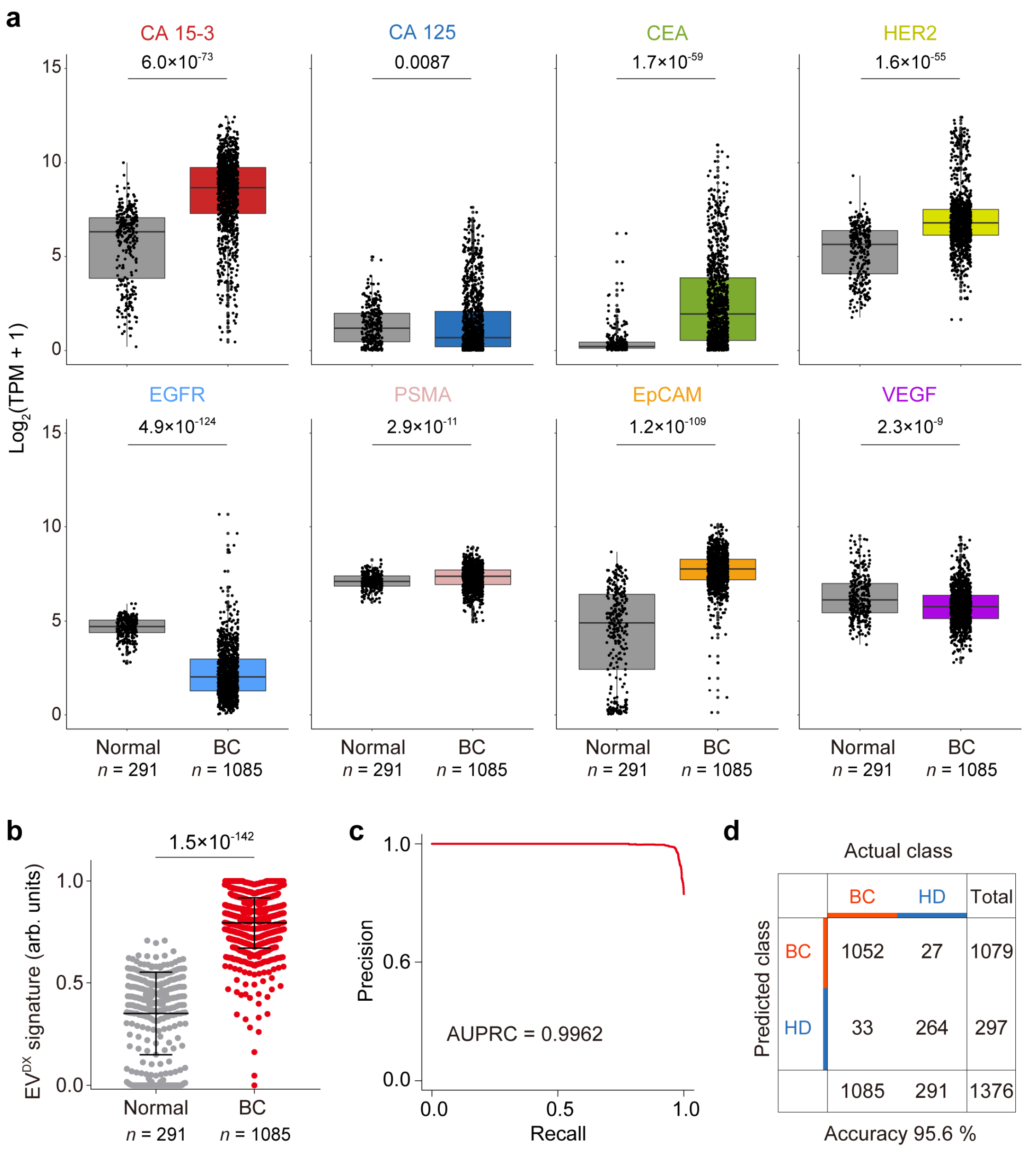

Supplementary Fig. 17 | Assessment of expression levels of mRNA transcripts of 8 protein markers in the public database. a, The analyses of breast carcinoma tissues (*n* = 1085, color box) and normal tissues (*n* = 291, gray box) based on The Cancer Genome Atlas (TCGA) and Genotype-Tissue Expression (GTEx) datasets by Gene Expression Profiling Interactive Analysis (GEPIA). The center lines of boxes represent the median

values, the up and down bounds of boxes represent the first quartile (Q1) and third quartile (Q3) values, and the minima and maxima of whiskers represent the Q1 − 1.5× IQR (interquartile range, Q3 − Q1) and Q3 + 1.5 × IQR. TPM, Transcripts Per Kilobase Million. **b-c,** Values (**b**) and PRC (**c**) of the signature (the weighted sum of mRNA transcript levels of the 8 markers by LDA). Red dots represent the signature for BC (n = 1085) and gray dots for normal tissue (n = 291) in (**b**). **d**, Confusion matrix showing an accuracy of 95.6 % for BC versus normal discrimination. Error bars represent the mean ± s.d. in (**b**). Statistical difference was determined by two-sided, nonparametric Mann-Whitney test (**a**,**b**). Source data are provided as a Source Data file.

Supplementary Table 1. Signaling, functions, and pathways of the 8 protein markers.

Protein	Signaling pathways	Functions	Reference
**CA 15-3**	PI3K-AKT<br>MEK-ERK	Survival and migration	Ref [1]
**CA 125**	JAK-STAT	Cell proliferation	Ref [2,3]
**CEA**	TGF-β	EMT and metastasis	Ref [4]
**HER2 & EGFR**	RAS-ERK, PI3K-AKT, and mTOR	Enhance proliferation, survival, and invasion	Ref [5]
**PSMA**	Integrin pathway	Neovasculature	Ref [6]
**EpCAM**	Wnt	Cell cycle and enhance cell stemness	Ref [7]
**VEGF**	VEGF signaling pathway	Angiogenesis and vascular permeability	Ref [8]

Supplementary Table 2. Summary of aptamers.

Aptamer	Sequence (5'-3')
**CA 15-3**	GCA GTT GAT CCT TTG GAT ACC CTG G
**CA 125**	TAT CAA TTA CTT ACC CTA GTG GTG TGA TGT CGT ATG GAT G
**CEA**	TTA ACT TAT TCG ACC ATA
**HER2**	GGG CCG TCG AAC ACG AGC ATG GTG CGT GGA CCT AGG ATG ACC TGA GTA CTG TCC
**EGFR**	TAC CAG TGC GAT GCT CAG TGC CGT TTC TTC TCT TTC GCT TTT TTT GCT TTT GAG CAT GCT GAC GCA TTC GGT TGA C
**PSMA**	GCG TTT TCG CTT TTG CGT TTT GGG TCA TCT GCT TAC GAT AGC AAT GCT
**EpCAM**	CAC TAC AGA GGT TGC GTC TGT CCC ACG TTG TCA TGG GGG GTT GGC CTG
**VEGF**	TTC CCG TCT TCC AGA CAA GAG TGC AGG G

Supplementary Table 3. Summary of aptamer properties.

Aptamer	Affinity	Specificity	Reference
**CA 15-3**	K_d = 0.135 nM	Discrimination of variations down to single amino acid changes	Ref [9]
**CA 125**	–	Binds to CA 125 and does not to CEA	Ref [10]
**CEA**	K_d = 5 nM	2 order of magnitude lower affinity for BSA; discrimination between CEA and other CEACAM family members	Ref [11]
**HER2**	K_d = 270 nM	2 order of magnitude lower affinity for BSA	Ref [12]
**EGFR**	K_d = 56 nM	Recognizes EGFR-positive cells (A431, U251, and U87) but does not bind to EGFR-negative cells (Jurkat).	Ref [13]
**PSMA**	–	Recognize PSMA-positive C4-2 cells but not bind to PSMA-negative PC-3 cells	Ref [14]
**EpCAM**	K_d = 22.8 nM	EpCAM+ Kato III; EpCAM- HEK-293T	Ref [15]
**VEGF**	K_d = ~400 nM	–	Ref [16]

Supplementary Table 4. Summary of MBC detection cohort.

Characteristic	MBC	NMBC	HD	Total
**Total cases**	36	21	66	123
**Subtypes**				
**HR+**	20	12	–	32
**HER2+**	8	8	–	16
**TNBC**	8	1	–	9
**Age (year)**				
**Median (range)**	56 (33 – 83)	55 (32 – 72)	51 (27 – 73)	52 (27 – 83)
**Tumor size at first diagnosis**				
**T1**	5	2	–	7
**T2**	13	10	–	23
**T3**	4	2	–	6
**T4**	3	1	–	4
**Unknown**	11	6	–	17
**Nodal status at first diagnosis**				
**Node negative**	8	3	–	11
**N1**	12	8	–	20
**N2**	5	0	–	5
**N3**	3	4	–	7
**Unknown**	8	6	–	14
**Tumor grade at first diagnosis**				
**I**	0	1	–	1
**II**	14	8	–	22
**III**	9	1	–	10
**Unknown**	13	11	–	24
**Stage at first diagnosis**				
**I**	4	2	–	6
**II**	16	10	–	26
**III**	6	6	–	12
**IV**	6	0	–	6
**Unknown**	4	3	–	7
**Sampling timing (day)**				
**Post to diagnosis of MBC/NMBC: median (range)**	6 (0 – 34)	10 (0 – 34)	–	8 (0 – 34)
**Prior to neoadjuvant/salvage treatment: median (range)**	2 (0 – 8)	3 (0 – 13)	–	2 (0 – 13)
**Post to surgery of primary tumor: median (range)**	1342 (62 – 5159)	–	–	1342 (62 – 5159)
**Plasma CA 15-3 (U mL^{-1})**				
**Median**	42.2	9.1	7.7	9.7
**Range**	5.7 – 371.8	5.7 – 79.3	3.8 – 46.5	3.8 – 371.8

Supplementary Table 5. The area under the Precision-Recall curves (AUPRC) for individual EV markers, SUM, and the EV^{DX} signature in BC versus HD discrimination as well as MBC versus NMBC discrimination.

	**BC versus HD (*n* = 123)**	**MBC versus NMBC (*n* = 57)**
**Markers**	AUPRC	AUPRC
**CA 15-3**	0.9286	0.7374
**CA 125**	0.8479	0.9068
**CEA**	0.8990	0.7458
**HER2**	0.8612	0.7833
**EGFR**	0.7554	0.7663
**PSMA**	0.8598	0.8631
**EpCAM**	0.9709	0.6704
**VEGF**	0.8796	0.7232
**SUM**	0.9825	0.8671
**EV^{DX} signature**	0.9912	0.9433

Supplementary Table 6. Performance of individual EV markers, SUM and the EV^{DX} signature in BC versus HD discrimination (Ninety-five percent CIs are indicated in parentheses).

**Markers**	**BC versus HD (*n* = 123)**			
	Sensitivity (%)	Specificity (%)	Accuracy (%)	AUC
EV CA 15-3	84.2<br>(72.1 – 92.5)	87.9<br>(77.5 – 94.6)	86.2<br>(78.8 – 91.7)	0.9254<br>(0.8764 – 0.9745)
EV CA 125	80.7<br>(68.1 – 90.0)	78.8<br>(67.0 – 87.9)	79.7<br>(71.5 – 86.4)	0.8320<br>(0.7553 – 0.9087)
EV CEA	84.2<br>(72.1 – 92.5)	78.8<br>(67.0 – 87.9)	81.3<br>(73.3 – 87.8)	0.8971<br>(0.8440 – 0.9503)
EV HER2	70.8<br>(56.6 – 81.6)	86.4<br>(75.7 – 93.6)	78.9<br>(70.6 – 85.7)	0.8612<br>(0.7948 – 0.9277)
EV EGFR	68.4<br>(54.8 – 80.1)	75.8<br>(63.6 – 85.5)	72.4<br>(63.6 – 80.0)	0.7770<br>(0.6966 – 0.8573)
EV PSMA	80.7<br>(68.1 – 90.0)	80.3<br>(68.7 – 89.1)	80.5<br>(72.4 – 87.1)	0.8591<br>(0.7935 – 0.9247)
EV EpCAM	94.7<br>(85.4 – 98.9)	90.9<br>(81.3 – 96.6)	92.7<br>(86.6 – 96.6)	0.9716<br>(0.9468 – 0.9963)
EV VEGF	86.0<br>(74.2 – 93.7)	78.8<br>(67.0 – 87.9)	82.1<br>(74.18 – 88.4)	0.9009<br>(0.8490 – 0.9527)
SUM	89.5<br>(78.5 – 96.0)	100.0<br>(94.6 – 100.0)	95.1<br>(89.7 – 98.2)	0.9814<br>(0.9626 – 1.0000)
EV signature	93.0<br>(83.0 – 98.1)	100.0<br>(94.6 – 100.0)	96.8<br>(92.0 – 99.1)	0.9918<br>(0.9822 – 1.0000)

Supplementary Table 7. Performance of individual EV markers, SUM and the EV^{DX} signature in MBC versus NMBC discrimination (Ninety-five percent CIs are indicated in parentheses).

**Markers**	**MBC versus NMBC (*n* = 57)**			
	Sensitivity (%)	Specificity (%)	Accuracy (%)	AUC
EV CA 15-3	61.1<br>(43.5 – 76.9)	85.7<br>(63.7 – 97.0)	70.2<br>(56.6 – 81.6)	0.6997<br>(0.5524 – 0.8470)
EV CA 125	91.7<br>(77.5 – 98.3)	71.4<br>(47.8 – 88.7)	84.2<br>(72.1 – 92.5)	0.8598<br>(0.7583 – 0.9613)
EV CEA	58.3<br>(40.8 – 74.5)	71.4<br>(47.8 – 88.7)	63.2<br>(49.3 – 75.6)	0.5926<br>(0.4418 – 0.7434)
EV HER2	75.0<br>(57.8 – 87.9)	47.6<br>(25.7 – 70.2)	64.9<br>(51.1 – 77.1)	0.6310<br>(0.4780 – 0.7749)
EV EGFR	66.7<br>(49.0 – 81.4)	76.2<br>(52.8 – 91.8)	70.2<br>(56.6 – 81.6)	0.6323<br>(0.4836 – 0.7810)
EV PSMA	63.9<br>(46.2 – 79.2)	81.0<br>(58.1 – 94.6)	70.2<br>(56.6 – 81.6)	0.7315<br>(0.6036 – 0.8593)
EV EpCAM	69.4<br>(51.9 – 83.7)	47.6<br>(25.7 – 70.2)	61.4<br>(47.6 – 74.0)	0.5939<br>(0.4353 – 0.7525)
EV VEGF	66.7<br>(49.0 – 81.4)	52.4<br>(29.8 – 87.9)	61.4<br>(47.6 – 74.0)	0.6019<br>(0.4508 – 0.7529)
SUM	69.4<br>(51.9 – 83.7)	81.0<br>(58.1 – 94.6)	73.7<br>(60.3 – 84.5)	0.7844<br>(0.6633 – 0.9055)
EV signature	94.4<br>(81.3 – 99.3)	76.2<br>(52.8 – 91.8)	87.7<br>(76.3 – 94.9)	0.9114<br>(0.8340 – 0.9888)

Supplementary Table 8. Performance of EV signatures derived from 7 EV markers in discrimination of MBC, NMBC and HD (Ninety-five percent CIs are indicated in parentheses).

**Markers**	**BC versus HD** Accuracy (%)	**MBC versus NMBC** Accuracy (%)	**Overall** Accuracy (%)
Without EV CA 15-3	95.1 (89.7 – 98.2)	86.0 (87.6 – 97.2)	88.6
Without EV CA 125	95.9 (90.8 – 98.7)	79.8 (69.2 – 88.0)	82.9
Without EV CEA	95.9 (90.8 – 98.7)	87.7 (76.3 – 94.9)	90.2
Without EV HER2	95.9 (90.8 – 98.7)	80.7 (68.1 – 90.0)	87.0
Without EV EGFR	95.9 (90.8 – 98.7)	82.5 (70.1 – 91.3)	87.8
Without EV PSMA	95.1 (89.7 – 98.2)	84.2 (72.1 – 92.5)	87.8
Without EV EpCAM	93.5 (87.6 – 97.2)	82.5 (70.1 – 91.3)	85.3
Without EV VEGF	95.9 (90.8 – 98.7)	84.2 (72.1 – 92.5)	88.6

Supplementary Table 9. Summary of training and validation cohorts for MBC monitoring.

Characteristic	PD	SD	PR	Total
**Total cases**	17	30	31	78
**Subtypes**				
**HR+**	5	9	8	22
**HER2+**	6	14	11	31
**TNBC**	6	7	12	25
**Age (year)**				
**Median (range)**	48 (31 – 80)	56 (37 – 80)	55 (28 – 80)	56 (28 – 80)
**Tumor size at first diagnosis**				
**T1**	8	4	5	17
**T2**	2	17	14	33
**T3**	4	2	3	9
**T4**	3	4	4	11
**Unknown**	0	3	5	8
**Nodal status at first diagnosis**				
**Node negative**	3	8	7	18
**N1**	4	8	14	26
**N2**	7	6	3	16
**N3**	3	6	4	13
**Unknown**	0	2	3	5
**Tumor grade at first diagnosis**				
**I**	0	1	0	1
**II**	10	11	12	33
**III**	5	6	6	17
**Unknown**	2	12	13	27
**Stage at first diagnosis**				
**I**	2	0	1	3
**II**	3	15	18	36
**III**	8	7	6	21
**IV**	4	8	5	17
**Unknown**	0	0	1	1
**Plasma CA15-3 (U mL^{-1})**				
**Median**	27.8	20.6	17.7	20.3
**Range**	6.8 – 1130	8.0 – 451	5.7 – 43.3	5.7 – 1130

Supplementary Table 10. Performance of EV markers in PR/SD versus PD discrimination for MBC patients across the training and validation cohorts (Ninety-five percent CIs are indicated in parentheses).

Markers	PR/SD versus PD (*n* = 78)			
	Sensitivity (%)	Specificity (%)	Accuracy (%)	AUC
EV CA15-3	64.7<br>(38.3 – 85.8)	67.1<br>(54.0 – 78.7)	66.7<br>(55.1 – 76.9)	0.7040<br>(0.5818 – 0.8261)
EV CA125	82.4<br>(56.6 – 96.2)	49.2<br>(36.1 – 62.3)	56.4<br>(44.7 – 67.6)	0.6673<br>(0.5302 – 0.8044)
EV CEA	70.6<br>(44.0 – 89.7)	86.9<br>(75.8 – 94.2)	83.3<br>(73.2 – 90.8)	0.8206<br>(0.6995 – 0.9418)
EV HER2	58.8<br>(32.9 – 81.6)	63.9<br>(50.6 – 75.8)	62.8<br>(51.1 – 73.5)	0.6905<br>(0.5646 – 0.8163)
EV EGFR	58.8<br>(32.9 – 81.6)	50.8<br>(37.7 – 63.9)	52.6<br>(40.9 – 64.0)	0.5429<br>(0.3880 – 0.6979)
EV PSMA	76.5<br>(50.1 – 93.2)	88.5<br>(77.8 – 95.3)	85.9<br>(76.2 – 92.7)	0.8447<br>(0.7335 – 0.9560)
EV EpCAM	70.6<br>(44.0 – 89.7)	78.7<br>(66.3 – 88.1)	76.9<br>(66.0 – 85.7)	0.7753<br>(0.6479 – 0.9028)
EV VEGF	64.7<br>(38.3 – 85.8)	75.4<br>(62.7 – 85.5)	73.1<br>(61.8 – 82.5)	0.7825<br>(0.6814 – 0.8836)
EV signature	76.5<br>(50.1 – 93.2)	91.8<br>(81.9 – 97.3)	88.5<br>(79.2 – 94.6)	0.9248<br>(0.8597 – 0.9899)

Supplementary Table 11. Summary of prospective cohort of MBC patients for treatment response monitoring.

Characteristic	PD	SD	PR	Total
**Total cases**	7	9	11	27
**Subtypes**				
**HR+**	4	4	5	13
**HER2+**	2	3	5	10
**TNBC**	1	2	1	4
**Age (year)**				
**Median (range)**	54 (43 – 58)	51 (43 – 63)	49 (38 – 80)	49 (38 – 80)
**Tumor size at first diagnosis**				
**T1**	0	2	1	3
**T2**	3	6	3	12
**T3**	0	0	1	1
**T4**	1	0	4	5
**Unknown**	3	1	2	6
**Nodal status at first diagnosis**				
**Node negative**	2	3	4	9
**N1**	1	2	4	7
**N2**	1	3	1	5
**N3**	0	0	0	0
**Unknown**	3	1	2	6
**Tumor grade at first diagnosis**				
**I**	0	0	0	0
**II**	1	3	2	6
**III**	2	2	3	7
**Unknown**	4	4	6	14
**Stage at first diagnosis**				
**I**	0	1	0	1
**II**	4	6	3	13
**III**	0	2	2	4
**IV**	2	0	4	6
**Unknown**	1	0	2	3
**Plasma CA15-3 (U mL^{-1})**				
**Median**	42.3	37.3	16.2	24.9
**Range**	12 – 1482	5.9 – 1036	4.5 – 115	4.5 – 1482

Supplementary Table 12. Performance of EV markers in PR/SD versus PD discrimination for MBC patients in a prospective cohort (Ninety-five percent CIs are indicated in parentheses).

**Markers**	**PR/SD versus PD (*n* = 27)**			
	Sensitivity (%)	Specificity (%)	Accuracy (%)	AUC
EV CA15-3	42.9<br>(9.9 – 81.6)	60.0<br>(36.1 – 80.9)	55.6<br>(35.3 – 74.5)	0.5929<br>(0.3620 – 0.8237)
EV CA125	57.1<br>(18.4 – 90.1)	60.0<br>(36.1 – 80.9)	59.3<br>(38.8 – 77.6)	0.5857<br>(0.2676 – 0.9038)
EV CEA	71.4<br>(29.0 – 96.3)	70.0<br>(45.7 – 88.1)	70.4<br>(49.8 – 86.3)	0.7429<br>(0.5423 – 0.9434)
EV HER2	85.7<br>(42.1 – 99.6)	59.1<br>(36.4 – 79.3)	65.5<br>(45.7 – 82.1)	0.6857<br>(0.4775 – 0.8939)
EV EGFR	42.9<br>(9.9 – 81.6)	59.1<br>(36.4 – 79.3)	55.2<br>(51.9 – 71.3)	0.6071<br>(0.3594 – 0.8549)
EV PSMA	71.4<br>(29.0 – 96.3)	75.0<br>(50.9 – 91.3)	74.1<br>(53.7 – 88.9)	0.8143<br>(0.6123 – 1.0000)
EV EpCAM	57.1<br>(18.4 – 90.1)	70.0<br>(45.7 – 88.1)	66.7<br>(46.0 – 83.5)	0.5429<br>(0.2385 – 0.8472)
EV VEGF	71.4<br>(29.0 – 96.3)	75.0<br>(50.9 – 91.3)	74.1<br>(53.7 – 88.9)	0.8000<br>(0.6236 – 0.9674)
EV signature	85.7<br>(42.1 – 99.6)	85.0<br>(62.1 – 96.8)	85.2<br>(66.3 – 95.8)	0.8929<br>(0.7542 – 1.0000)

Supplementary Table 13. Summary of MBC prognosis cohort.

Characteristic	PD	Censored	Total
**Total cases**	14	45	59
**Subtypes**			
**HR+**	3	19	22
**HER2+**	5	19	24
**TNBC**	6	7	13
**Age**			
**Median**	56	52	55
**Range**	34 – 80	28 – 70	28 – 80
**Baseline Plasma CA15-3 (U mL^{-1})**			
**Median**	20.3	24.8	22.5
**Range**	10.1 – 202.4	5.6 – 756.5	5.6 –756.5

Supplementary Table 14. Summary of prognostic cohort of MBC prognosis.

Characteristic	PD	Censored	Total
**Total cases**	5	11	16
**Subtypes**			
**HR+**	4	3	7
**HER2+**	0	5	5
**TNBC**	1	3	4
**Age**			
**Median**	43	47	46
**Range**	37 – 48	30 – 69	30 – 69
**Baseline Plasma CA15-3 (U mL^{-1})**			
**Median**	58.9	16.5	18.8
**Range**	4.6 – 546	5.5 – 103	4.6 – 546

Supplementary Table 15. The cost of TAS for detection of one individual.

	Quantity	Unit price	Cost
Aptamer	80 pmol	0.006 \$ $pmol^{-1}$	0.48 \$
Glass slide	8	0.03 \$	0.24 \$
Tape	~ 0.5 m	0.014 \$	~0.007 \$
Pipette tip	12	0.01 \$	0.12 \$
Total	—	—	0.85 \$

Supplementary Table 16. Comparison of LDA and logistic regression (LR) classifiers in BC versus HD discrimination, MBC versus NMBC discrimination, and PR/SD versus PD discrimination (Ninety-five percent CIs are indicated in parentheses).

**BC versus HD (*n* = 123)**	**LDA**	**LR**
**Sensitivity (%)**	94.8 (85.4 – 98.9)	96.6 (88.1 – 99.6)
**Specificity (%)**	100.0 (94.6 – 100.0)	98.5 (91.8 – 100.0)
**Accuracy (%)**	97.6 (93.0 – 99.5)	97.6 (93.0 – 99.5)
**AUC**	0.9918 (0.9822 – 1.0000)	0.9957 (0.9892 – 1.0000)
**MBC versus NMBC (*n* = 57)**	LDA	LR
**Sensitivity (%)**	94.4 (81.3 – 99.3)	91.7 (77.5 – 98.3)
**Specificity (%)**	76.2 (52.8 – 91.8)	76.2 (52.8 – 91.8)
**Accuracy (%)**	87.7 (76.3 – 94.9)	86.0 (74.2 – 93.7)
**AUC**	0.9114 (0.8340 – 0.9888)	0.9140 (0.8399 – 0.9882)
**PR/SD versus PD (*n* = 78)**	LDA	LR
**Sensitivity (%)**	76.5 (50.1 – 93.2)	82.4 (56.6 – 96.2)
**Specificity (%)**	91.8 (81.9 – 97.3)	91.8 (81.9 – 97.3)
**Accuracy (%)**	88.5 (79.2 – 94.6)	89.7 (80.8 – 95.5)
**AUC**	0.9248 (0.8597 – 0.9899)	0.8944 (0.8000 – 0.9888)

References

1 Kufe, D. W. MUC1-C oncoprotein as a target in breast cancer: activation of signaling pathways and therapeutic approaches. *Oncogene* **32**, 1073 (2012).

2 Reinartz, S., Failer, S., Schuell, T. & Wagner, U. CA125 (MUC16) gene silencing suppresses growth properties of ovarian and breast cancer cells. *Eur. J. Cancer* **48**, 1558-1569 (2012).

3 Lakshmanan, I. *et al.* MUC16 induced rapid G2/M transition via interactions with JAK2 for increased proliferation and anti-apoptosis in breast cancer cells. *Oncogene* **31**, 805-817 (2012).

4 Powell, E. *et al.* A functional genomic screen in vivo identifies CEACAM5 as a clinically relevant driver of breast cancer metastasis. *npj Breast Cancer* **4**, 9 (2018).

5 Tebbutt, N., Pedersen, M. W. & Johns, T. G. Targeting the ERBB family in cancer: couples therapy. *Nat. Rev. Cancer* **13**, 663-673 (2013).

6 Conway, R. E. *et al.* Prostate-Specific Membrane Antigen Regulates Angiogenesis by Modulating Integrin Signal Transduction. *Mol. Cell. Biol.* **26**, 5310-5324 (2006).

7 Munz, M., Baeuerle, P. A. & Gires, O. The Emerging Role of EpCAM in Cancer and Stem Cell Signaling. *Cancer Res.* **69**, 5627-5629 (2009).

8 Goel, H. L. & Mercurio, A. M. VEGF targets the tumour cell. *Nat. Rev. Cancer* **13**, 871-882 (2013).

9 Ferreira, C. S. M., Matthews, C. S. & Missailidis, S. DNA Aptamers That Bind to

MUC1 Tumour Marker: Design and Characterization of MUC1-Binding Single-Stranded DNA Aptamers. *Tumor Biol.* **27**, 289-301 (2006).

10 Gedi, V. *et al.* Sensitive on-chip detection of cancer antigen 125 using a DNA aptamer/carbon nanotube network platform. *Sens. Actuator B-Chem.* **256**, 89-97 (2018).

11 Smith, C. L. Compositions comprising nucleic acid aptamers. U.S. patent US20130101506A1 (2006).

12 Niazi, J. H., Verma, S. K., Niazi, S. & Qureshi, A. In vitro HER2 protein-induced affinity dissociation of carbon nanotube-wrapped anti-HER2 aptamers for HER2 protein detection. *Analyst* **140**, 243-249 (2015).

13 Wang, D.-L. *et al.* Selection of DNA aptamers against epidermal growth factor receptor with high affinity and specificity. *Biochem. Biophys. Res. Commun.* **453**, 681-685 (2014).

14 Boyacioglu, O., Stuart, C. H., Kulik, G. & Gmeiner, W. H. Dimeric DNA Aptamer Complexes for High-capacity-targeted Drug Delivery Using pH-sensitive Covalent Linkages. *Mol. Ther.-Nucl. Acids* **2** (2013).

15 Song, Y. *et al.* Selection of DNA Aptamers against Epithelial Cell Adhesion Molecule for Cancer Cell Imaging and Circulating Tumor Cell Capture. *Anal. Chem.* **85**, 4141-4149 (2013).

16 Potty, A. S. R. *et al.* Biophysical characterization of DNA aptamer interactions with vascular endothelial growth factor. *Biopolymers* **91**, 145-156 (2009).
