# Supplementary material for: Protein analysis of extracellular vesicles to monitor and predict therapeutic response in metastatic breast cancer
Source: Nat Commun. 2021 May 5;12:2536. doi: 10.1038/s41467-021-22913-7 (PMC8100127; doi:10.1038/s41467-021-22913-7)
Supplement: Supplementary file 3 — Description of Additional Supplementary Files [file 41467_2021_22913_MOESM3_ESM.docx]

Description of Additional Supplementary Files

Title: Supplementary Data 1

Description: Additional patient information

Title: Supplementary Software

Description: Code for linear discriminant analysis
