# Supplementary material for: Protein analysis of extracellular vesicles to monitor and predict therapeutic response in metastatic breast cancer
Source: Nat Commun. 2021 May 5;12:2536. doi: 10.1038/s41467-021-22913-7 (PMC8100127; doi:10.1038/s41467-021-22913-7)
Supplement: Supplementary file 4 — Supplementary Data 1 [file 41467_2021_22913_MOESM4_ESM.zip › NCOMMS-20-44503C-Supplementary-Data-1.pdf]

Supplementary Data. Additional patient information. ▲ indicate the plasma sampling.

Patient	Status	Gender	Age	Subtype	Baseline	Treatment-1	Treatment-2	Treatment-3	Treatment-4
2	MBC	Female	33	HR+ HER2-	Post-surgery, 8 weeks<br>MBC recurrence, 0 day<br>Prior to salvage therapy▲				
15	MBC	Female	37	HR+ HER2-	MBC diagnosis, 4 weeks<br>Prior to salvage therapy▲	paclitaxel + capecitabine<br>6 weeks, PR▲	paclitaxel + capecitabine<br>6 weeks, SD▲		
26	MBC	Female	63	HR+ HER2-	Post-surgery, 362 weeks<br>MBC recurrence, 4 weeks<br>Prior to salvage therapy▲	paclitaxel + capecitabine<br>4 weeks, SD▲	paclitaxel + capecitabine<br>4 weeks, SD▲	paclitaxel + capecitabine<br>6 weeks, SD▲	paclitaxel + capecitabine<br>6 weeks, PR▲
29	MBC	Female	50	HR+ HER2-	MBC recurrence, 0 day<br>Prior to salvage therapy▲	paclitaxel<br>14 weeks, SD▲			
35	MBC	Female	48	HR+ HER2-	MBC recurrence, 0 day<br>Prior to salvage therapy▲	letrozole + leuprorelin<br>32 weeks, SD▲			
38	MBC	Female	70	HR+ HER2-	Prior to salvage therapy▲	fulvestrant<br>7 weeks, PR▲			
39	MBC	Female	72	HR+ HER2-	Prior to salvage therapy▲	fulvestrant<br>11 weeks, PR▲			
49	MBC	Female	49	HR+ HER2-	Post-surgery, 527 weeks<br>MBC recurrence, 0 day<br>Prior to salvage therapy▲				
50	MBC	Female	60	HR+ HER2-	Post-surgery, 737 weeks<br>MBC recurrence, 12 days<br>Prior to salvage therapy▲	fulvestrant<br>9 weeks, PR▲			
51	MBC	Female	26	HR+ HER2-	Prior to salvage therapy<br>Prior to salvage therapy▲	letrozole + palbociclib<br>9 weeks, SD▲	letrozole + palbociclib<br>14 weeks, SD		
56	NMBC	Female	61	HR+ HER2-	NMBC diagnosis, 15 days<br>Prior to neoadjuvant therapy▲				
60	MBC	Female	46	HR+ HER2-	MBC diagnosis, 28 days<br>Prior to salvage therapy▲	paclitaxel + capecitabine<br>7 weeks, PR▲	paclitaxel + capecitabine<br>6 weeks, PR▲		
63	MBC	Female	56	HR+ HER2-	Prior to salvage therapy▲	apatinib			

						11 weeks, PD▲			
74	NMBC	Female	64	HR+ HER2-	NMBC diagnosis, 34 days<br>Prior to neoadjuvant therapy▲				
76	MBC	Female	45	HR+ HER2-	MBC diagnosis, 5 days<br>Prior to salvage therapy▲	paclitaxel + zoledronic acid<br>5 weeks, PR▲			
85	MBC	Female	48	HR+ HER2-	Post-surgery, 458 weeks<br>MBC recurrence, 7 days<br>Prior to salvage therapy▲	paclitaxel<br>8 weeks, PR▲	paclitaxel + capecitabine<br>12 weeks, PR▲	capecitabine<br>11 weeks, PD▲	
86	MBC	Female	38	HR+ HER2-	Post-surgery, 77 weeks<br>MBC recurrence, 1 day<br>Prior to salvage therapy▲	paclitaxel + doxorubicin<br>7 weeks, SD▲	paclitaxel + doxorubicin<br>15 weeks, PR▲		
89	MBC	Female	33	HR+ HER2-	Prior to salvage therapy▲	vinorelbine + pyrotinib<br>9 weeks, PD▲	paclitaxel<br>6 weeks, PD▲	apatinib<br>13 weeks, PD▲	
90	MBC	Female	43	HR+ HER2-	Prior to salvage therapy▲	goserelin + letrozole + palbociclib<br>7 weeks, SD▲	goserelin + letrozole + palbociclib<br>5 weeks, PD▲	bevacizumab + vinorelbine<br>5 weeks, SD▲	
92	NMBC	Female	51	HR+ HER2-	NMBC diagnosis, 6 days<br>Prior to neoadjuvant therapy▲				
96	MBC	Female	65	HR+ HER2-	Post-surgery, 413 weeks<br>MBC recurrence, 0 day<br>Prior to salvage therapy▲				
97	MBC	Female	38	HR+ HER2-	Prior to salvage therapy▲	paclitaxel<br>6 weeks, PD▲			
100	MBC	Female	61	HR+ HER2-	Post-surgery, 549 weeks<br>MBC recurrence, 0 day<br>Prior to salvage therapy▲	paclitaxel<br>7 weeks, SD▲	paclitaxel<br>24 week, SD		
102	MBC	Female	56	HR+ HER2-	Prior to salvage therapy▲	fulvestrant<br>26 weeks, SD			
111	MBC	Female	56	HR+ HER2-	MBC diagnosis, 22 days<br>Prior to salvage therapy▲	paclitaxel + capecitabine<br>7 weeks, SD▲	paclitaxel + capecitabine<br>7 weeks, SD		
114	MBC	Female	47	HR+ HER2-	Prior to salvage therapy▲	paclitaxel<br>7 weeks, SD▲	anastrozole + goserelin<br>68 weeks, PR		
116	MBC	Female	43	HR+ HER2-	Prior to salvage therapy▲	capecitabine	capecitabine		

						25 weeks, PR▲	7 weeks, PD▲		
122	MBC	Female	46	HR+ HER2-	Post-surgery, 100 weeks<br>MBC recurrence, 22 days<br>Prior to salvage therapy▲	anastrozole + leuprorelin<br>6 weeks, SD▲	anastrozole + leuprorelin<br>22 weeks, SD		
127	MBC	Female	65	HR+ HER2-	Post-surgery, 563 weeks<br>MBC recurrence, 26 days<br>Prior to salvage therapy▲				
132	MBC	Female	47	HR+ HER2-	Prior to salvage therapy▲	paclitaxel + capecitabine<br>23 weeks, SD			
133	NMBC	Female	32	HR+ HER2-	NMBC diagnosis, 24 days<br>Prior to neoadjuvant therapy▲				
155	MBC	Female	52	HR+ HER2-	Post-surgery, 159 weeks<br>MBC recurrence, 13 days<br>Prior to salvage therapy▲	anastrozole + goserelin<br>55 weeks, PD			
159	MBC	Female	38	HR+ HER2-	Post-surgery, 154 weeks<br>MBC recurrence, 17 days<br>Prior to salvage therapy▲				
164	MBC	Female	64	HR+ HER2-	Post-surgery, 531 weeks<br>MBC recurrence, 2 days<br>Prior to salvage therapy▲				
165	MBC	Female	68	HR+ HER2-	Prior to salvage therapy▲	paclitaxel<br>14 weeks, SD			
168	NMBC	Female	62	HR+ HER2-	NMBC diagnosis, 12 days<br>Prior to neoadjuvant therapy▲				
210	MBC	Female	64	HR+ HER2-	Prior to salvage therapy▲	paclitaxel<br>17 weeks, SD			
211	MBC	Female	47	HR+ HER2-	Post-surgery, 279 weeks<br>MBC recurrence, 3 days<br>Prior to salvage therapy▲	paclitaxel + capecitabine<br>9 weeks, SD	vinorelbine<br>15 weeks, SD		
271	NMBC	Female	55	HR+ HER2-	NMBC diagnosis, 15 days<br>Prior to neoadjuvant therapy▲				
288	MBC	Female	48	HR+ HER2-	Prior to salvage therapy▲	paclitaxel<br>13 weeks, SD	toremifene<br>28 weeks, PD		

344	MBC	Female	54	HR+ HER2-	Prior to salvage therapy▲	paclitaxel<br>6 weeks, PD▲			
345	NMBC	Female	40	HR+ HER2-	NMBC diagnosis, 2 days<br>Prior to neoadjuvant therapy▲				
357	NMBC	Female	46	HR+ HER2-	NMBC diagnosis, 0 day<br>Prior to neoadjuvant therapy▲				
414	NMBC	Female	52	HR+ HER2-	NMBC diagnosis, 7 days<br>Prior to neoadjuvant therapy▲				
415	NMBC	Female	32	HR+ HER2-	NMBC diagnosis, 14 days<br>Prior to neoadjuvant therapy▲				
449	MBC	Female	48	HR+ HER2-	Prior to salvage therapy▲	anastrozole + goserelin<br>7 weeks, PD			
503	MBC	Female	50	HR+ HER2-	Post-surgery, 86 weeks<br>MBC recurrence, 21 days<br>Prior to salvage therapy▲	paclitaxel<br>25 weeks, SD			
517	MBC	Female	37	HR+ HER2-	Prior to salvage therapy▲	fulvestrant + goserelin<br>12 weeks, PD			
531	NMBC	Female	49	HR+ HER2-	NMBC diagnosis, 0 day<br>Prior to neoadjuvant therapy▲	letrozole<br>7 weeks, SD			
581	MBC	Female	57	HR+ HER2-	Post-surgery, 615 weeks<br>MBC recurrence, 14 days<br>Prior to salvage therapy▲				
621	MBC	Female	43	HR+ HER2-	Prior to salvage therapy▲	paclitaxel<br>8 weeks, SD	vinorelbine<br>26 weeks, PD		
681	MBC	Female	41	HR+ HER2-	MBC diagnosis, 18 days<br>Prior to salvage therapy▲	paclitaxel<br>31 weeks, PR	anastrozole + leuprorelin<br>6 weeks, SD		
704	NMBC	Female	61	HR+ HER2-	NMBC diagnosis, 2 days<br>Prior to neoadjuvant therapy▲				
18	MBC	Female	56	HR+ HER2+	Prior to salvage therapy▲	capecitabine + trastuzumab<br>12 weeks, SD▲	capecitabine + trastuzumab<br>26 weeks, PR		
31	MBC	Female	59	HR+ HER2+	Prior to salvage therapy▲	anastrozole + trastuzumab<br>4 weeks, PD▲	capecitabine + pyrotinib<br>13 weeks, PR▲	capecitabine + pyrotinib<br>8 weeks, PD▲	
40	MBC	Female	48	HR+ HER2+	Prior to salvage therapy▲	anastrozole + goserelin +			

						lapatinib<br>16 weeks, SD			
46	MBC	Female	56	HR+ HER2+	Post-surgery, 192 weeks<br>MBC recurrence, 4 days<br>Prior to salvage therapy▲	fulvestrant + lapatinib<br>9 weeks, SD▲	fulvestrant + lapatinib<br>7 weeks, SD▲		
67	MBC	Female	32	HR+ HER2+	Prior to salvage therapy▲	capecitabine + lapatinib<br>13 weeks, SD			
94	MBC	Female	56	HR+ HER2+	Prior to salvage therapy▲	capecitabine + lapatinib<br>6 weeks, SD▲	capecitabine + lapatinib<br>18 weeks, SD		
124	MBC	Female	59	HR+ HER2-	Prior to salvage therapy▲	paclitaxel +<br>cyclophosphamide<br>11 weeks, PR▲	paclitaxel +<br>cyclophosphamide<br>4 weeks, PR▲		
142	MBC	Female	78	HR+ HER2+	Prior to salvage therapy▲	trastuzumab + lapatinib<br>4 weeks, SD			
154	MBC	Female	56	HR+ HER2+	Post-surgery, 135 weeks<br>MBC recurrence, 12 days<br>Prior to salvage therapy▲				
201	MBC	Female	54	HR+ HER2+	Prior to salvage therapy▲	trastuzumab<br>44 weeks, PR			
212	MBC	Female	19	HR+ HER2+	Prior to salvage therapy▲	anastrozole + goserelin +<br>trastuzumab + platinum<br>7 weeks, SD▲			
246	MBC	Female	43	HR+ HER2+	Prior to salvage therapy▲	pyrotinib<br>18 weeks, PD			
258	MBC	Female	52	HR+ HER2+	Prior to salvage therapy▲	paclitaxel + trastuzumab<br>17 weeks, PR▲	paclitaxel + trastuzumab<br>4 weeks, SD▲		
272	MBC	Female	44	HR+ HER2+	Prior to salvage therapy▲	paclitaxel + lapatinib<br>14 weeks, SD▲	paclitaxel + lapatinib<br>33 weeks, PD		
273	MBC	Female	50	HR+ HER2+	Prior to salvage therapy▲	paclitaxel + capecitabine +<br>trastuzumab<br>5 weeks, PR▲	capecitabine + trastuzumab,<br>12 weeks, SD		
278	MBC	Female	50	HR+ HER2+	Prior to salvage therapy▲	vinorelbine + platinum<br>4 weeks, SD▲	vinorelbine + platinum<br>17 weeks, PR		

362	MBC	Female	59	HR+ HER2+	Prior to salvage therapy▲	capecitabine + trastuzumab<br>36 weeks, SD			
366	NMBC	Female	62	HR+ HER2+	NMBC diagnosis, 13 days<br>Prior to neoadjuvant therapy▲				
400	MBC	Female	50	HR+ HER2+	Prior to salvage therapy▲	gemcitabine + trastuzumab<br>7 weeks, PD▲			
431	MBC	Female	47	HR+ HER2+	Prior to salvage therapy▲	apatinib<br>8 weeks, SD	capecitabine +apatinib<br>6 weeks, SD		
691	NMBC	Female	56	HR+ HER2+	NMBC diagnosis, 10 days<br>Prior to neoadjuvant therapy▲				
694	MBC	Female	30	HR+ HER2+	Prior to salvage therapy▲	capecitabine + pyrotinib<br>48 weeks, SD			
14	MBC	Female	46	HR- HER2+	Prior to salvage therapy▲	vinorelbine + pyrotinib<br>5 weeks, PD▲	vinorelbine + pyrotinib<br>9 weeks, SD▲		
47	MBC	Female	57	HR- HER2+	Prior to salvage therapy▲	capecitabine + trastuzumab + pertuzumab<br>8 weeks, PR▲	capecitabine + trastuzumab + pertuzumab<br>8 weeks, PR▲		
54	NMBC	Female	48	HR- HER2+	NMBC diagnosis, 9 days<br>Prior to neoadjuvant therapy▲				
73	NMBC	Female	47	HR- HER2+	NMBC diagnosis, 0 day<br>Prior to neoadjuvant therapy▲				
84	MBC	Female	56	HR- HER2+	MBC recurrence, 21 days<br>Prior to salvage therapy▲	paclitaxel + trastuzumab<br>6 weeks, SD▲	paclitaxel + trastuzumab<br>6 weeks, SD▲	paclitaxel + trastuzumab<br>13 weeks, SD	
112	MBC	Female	58	HR- HER2+	Prior to salvage therapy▲	capecitabine<br>7 weeks, PD▲	paclitaxel<br>6 weeks, SD▲	paclitaxel<br>16 weeks, SD	
117	MBC	Female	44	HR- HER2+	Prior to salvage therapy▲	paclitaxel + trastuzumab<br>12 weeks, PR	paclitaxel + trastuzumab<br>15 weeks, SD		
134	MBC	Female	64	HR- HER2+	Post-surgery, 54 weeks<br>MBC recurrence, 24 days<br>Prior to salvage therapy▲	capecitabine + pyrotinib<br>51 weeks, SD			
151	MBC	Female	49	HR- HER2+	Post-surgery, 204 weeks<br>MBC recurrence, 13 days<br>Prior to salvage therapy▲	paclitaxel + capecitabine + trastuzumab<br>23 weeks, PR▲			

175	MBC	Female	57	HR- HER2+	Prior to salvage therapy▲	vinorelbine + lapatinib<br>7 weeks, SD▲	vinorelbine + lapatinib<br>16 weeks, SD		
192	MBC	Female	56	HR- HER2+	Prior to salvage therapy▲	medroxyprogesterone + trastuzumab<br>5 weeks, SD▲	medroxyprogesterone + trastuzumab<br>11 weeks, PD▲	pyrotinib<br>11 weeks, SD▲	
216	MBC	Female	58	HR- HER2+	Prior to salvage therapy▲	paclitaxel + trastuzumab<br>17 weeks, PD▲	pyrotinib<br>8 weeks, PR▲		
274	MBC	Female	53	HR- HER2+	Prior to salvage therapy▲	paclitaxel + trastuzumab + platinum<br>5 weeks, PR▲			
289	NMBC	Female	67	HR- HER2+	NMBC diagnosis, 2 days<br>Prior to neoadjuvant therapy▲				
363	MBC	Female	49	HR- HER2+	Prior to salvage therapy▲	trastuzumab<br>7 weeks, PR▲	trastuzumab<br>29 weeks, SD		
368	MBC	Female	62	HR- HER2+	Prior to salvage therapy▲	capecitabine + pyrotinib<br>9 weeks, PR▲			
389	MBC	Female	58	HR- HER2+	Prior to salvage therapy▲	capecitabine + lapatinib<br>6 weeks, PR▲			
391	MBC	Female	46	HR- HER2+	MBC diagnosis, 6 days<br>Prior to salvage therapy▲	paclitaxel + capecitabine + trastuzumab<br>20 weeks, PR			
404	MBC	Female	63	HR- HER2+	Post-surgery, 163 weeks<br>MBC recurrence, 6 days<br>Prior to salvage therapy▲	capecitabine + pyrotinib<br>7 weeks, SD▲	capecitabine + pyrotinib<br>8 weeks, SD		
420	NMBC	Female	63	HR- HER2+	NMBC diagnosis, 6 days<br>Prior to neoadjuvant therapy▲				
433	MBC	Female	83	HR- HER2+	Post-surgery, 510 weeks<br>MBC recurrence, 34 days<br>Prior to salvage therapy▲				
453	NMBC	Female	46	HR- HER2+	NMBC diagnosis, 12 days<br>Prior to neoadjuvant therapy▲				
587	NMBC	Female	72	HR- HER2+	NMBC diagnosis, 4 days<br>Prior to neoadjuvant therapy▲				

34	MBC	Female	34	TNBC	Prior to salvage therapy▲	vinorelbine<br>20 weeks, PR▲	vinorelbine<br>16 weeks, PD▲		
45	MBC	Female	80	TNBC	MBC diagnosis, 6 days<br>Prior to salvage therapy▲	capecitabine<br>9 weeks, SD▲	capecitabine<br>6 weeks, SD▲	capecitabine<br>7 weeks, PD▲	paclitaxel<br>10 weeks, PR▲
57	MBC	Female	46	TNBC	Post-surgery, 107 weeks<br>MBC recurrence, 6 days<br>Prior to salvage therapy▲	vinorelbine + platinum<br>7 weeks, PR▲	vinorelbine + platinum<br>6 weeks, PR▲	vinorelbine<br>21 weeks, PR▲	
61	MBC	Female	51	TNBC	Post-surgery, 71 weeks<br>MBC recurrence, 0 day<br>Prior to salvage therapy▲	paclitaxel<br>6 weeks, PD▲			
64	MBC	Female	55	TNBC	Prior to salvage therapy▲	capecitabine + bevacizumab<br>5 weeks, PD▲			
66	MBC	Female	45	TNBC	Prior to salvage therapy▲	capecitabine<br>7 weeks, SD▲	capecitabine<br>7 weeks, SD▲		
68	NMBC	Female	41	TNBC	NMBC diagnosis, 27 days<br>Prior to neoadjuvant therapy▲				
82	MBC	Female	60	TNBC	Prior to salvage therapy▲	paclitaxel + toripalimab<br>7 weeks, SD▲	vinorelbine + platinum<br>7 weeks, PR▲		
88	MBC	Female	58	TNBC	Post-surgery, 164 weeks<br>MBC recurrence, 6 days<br>Prior to salvage therapy▲	paclitaxel<br>10 weeks, SD▲	paclitaxel<br>3 weeks, SD▲		
93	MBC	Female	70	TNBC	Post-surgery, 63 weeks<br>MBC recurrence, 6 days<br>Prior to salvage therapy▲	paclitaxel + toripalimab<br>10 weeks, PR▲	paclitaxel + toripalimab<br>3 weeks, PR▲	paclitaxel + toripalimab<br>7 week, PD	
101	MBC	Female	46	TNBC	MBC recurrence, 6 days<br>Prior to salvage therapy▲	paclitaxel + capecitabine<br>6 weeks, SD▲	paclitaxel + capecitabine<br>7 weeks, SD	paclitaxel<br>6 weeks, PD	
131	MBC	Female	55	TNBC	Prior to salvage therapy▲	apatinib<br>5 weeks, PR▲	apatinib<br>10 weeks, PD		
178	MBC	Female	46	TNBC	Prior to salvage therapy▲	paclitaxel + capecitabine<br>7 weeks, PR▲	paclitaxel + capecitabine<br>16 weeks, PR		
226	MBC	Female	62	TNBC	Prior to salvage therapy▲	apatinib<br>11 weeks, SD			
245	MBC	Female	28	TNBC	Prior to salvage therapy▲	vinorelbine + platinum	vinorelbine + platinum		

						6 weeks, PR▲	15 weeks, PR		
248	MBC	Female	31	TNBC	Prior to salvage therapy▲	etoposide<br>16 weeks, PD▲	gemcitabine<br>6 weeks, PD▲		
287	MBC	Female	51	TNBC	Prior to salvage therapy▲	paclitaxel<br>9 weeks, PD▲	apatinib<br>7 weeks, SD▲		
343	MBC	Female	78	TNBC	Post-surgery, 23 weeks<br>MBC recurrence, 0 day<br>Prior to salvage therapy▲				
384	MBC	Female	61	TNBC	Post-surgery, 450 weeks<br>MBC recurrence, 12 day<br>Prior to salvage therapy▲	paclitaxel + capecitabine<br>7 weeks, PR▲	paclitaxel + capecitabine<br>8 weeks, SD		
529	MBC	Female	37	TNBC	Post-surgery, 45 weeks<br>MBC recurrence, 20 days<br>Prior to salvage therapy▲	apatinib + toripalimab<br>38 weeks, PD			
548	MBC	Female	43	TNBC	Post-surgery, 414 weeks<br>MBC recurrence, 0 day<br>Prior to salvage therapy▲	paclitaxel + toripalimab<br>36 weeks, PD			
550	MBC	Female	35	TNBC	Post-surgery, 94 weeks<br>MBC recurrence, 0 day<br>Prior to salvage therapy▲	bevacizumab<br>13 weeks, PR			
585	MBC	Female	69	TNBC	Post-surgery, 625 weeks<br>MBC recurrence, 18 days<br>Prior to salvage therapy▲	paclitaxel<br>54 weeks, PR			
