# Supplementary material for: Protein analysis of extracellular vesicles to monitor and predict therapeutic response in metastatic breast cancer
Source: Nat Commun. 2021 May 5;12:2536. doi: 10.1038/s41467-021-22913-7 (PMC8100127; doi:10.1038/s41467-021-22913-7)
Supplement: Supplementary file 5 — Supplementary Software [file 41467_2021_22913_MOESM5_ESM.zip › Supplementary-Software/Readme.docx]

1. System requirements. The code has been tested using R4.0.1 on Windows 7 (64-bit).
2. Install R4.0.1 and RStudio 1.3.1093.
3. Open RStudio and load the code file.
4. Prepare CSV data files in Excel. Each row represents a patient and each patient has 10 columns for patient index, status (BC versus HD, MBC versus NMBC, PD versus PR/SD, or high versus low baseline expression), and expression levels or relative intensities of the 8 protein markers.
5. Put the data files to default path. Click “Run” on RStudio.
