# Supplementary material for: Protein analysis of extracellular vesicles to monitor and predict therapeutic response in metastatic breast cancer
Source: Nat Commun. 2021 May 5;12:2536. doi: 10.1038/s41467-021-22913-7 (PMC8100127; doi:10.1038/s41467-021-22913-7)
Supplement: Supplementary file 6 — Reporting Summary [file 41467_2021_22913_MOESM6_ESM.pdf]

nature research

Corresponding author(s): Jiashu Sun, Zefei Jiang, and Chao Liu

Last updated by author(s): Mar 26, 2021

Reporting Summary

Nature Research wishes to improve the reproducibility of the work that we publish. This form provides structure for consistency and transparency in reporting. For further information on Nature Research policies, see our Editorial Policies and the Editorial Policy Checklist.

Statistics

For all statistical analyses, confirm that the following items are present in the figure legend, table legend, main text, or Methods section.

n/a	Confirmed	
☐	☒	The exact sample size (n) for each experimental group/condition, given as a discrete number and unit of measurement
☐	☒	A statement on whether measurements were taken from distinct samples or whether the same sample was measured repeatedly
☐	☒	The statistical test(s) used AND whether they are one- or two-sided<br>*Only common tests should be described solely by name; describe more complex techniques in the Methods section.*
☒	☐	A description of all covariates tested
☒	☐	A description of any assumptions or corrections, such as tests of normality and adjustment for multiple comparisons
☐	☒	A full description of the statistical parameters including central tendency (e.g. means) or other basic estimates (e.g. regression coefficient) AND variation (e.g. standard deviation) or associated estimates of uncertainty (e.g. confidence intervals)
☐	☒	For null hypothesis testing, the test statistic (e.g. F, t, r) with confidence intervals, effect sizes, degrees of freedom and P value noted<br>*Give P values as exact values whenever suitable.*
☒	☐	For Bayesian analysis, information on the choice of priors and Markov chain Monte Carlo settings
☒	☐	For hierarchical and complex designs, identification of the appropriate level for tests and full reporting of outcomes
☐	☒	Estimates of effect sizes (e.g. Cohen's d, Pearson's r), indicating how they were calculated

Our web collection on statistics for biologists contains articles on many of the points above.

Software and code

Policy information about availability of computer code

Data collection	Fluorescence images were recorded using ImageJ 1.52a.
Data analysis	The NTA data were captured and analysed using the NTA 3.4 Analytical Software Suite. Fluorescence images were analyzed using ImageJ 1.52a. Significance analyses, Pearson correlation, ROC curve construction and AUC calculation were performed using GraphPad Prism 7. t-SNE were performed using Matlab 2015b. PRC construction, AUPRC calculation, hierarchical clustering, Kaplan-Meier analysis, log-rank test and Cox regression were performed using R software (version 4.0.1). LDA (based on package MASS, version 7.3) was performed using the custom codes written using R software (version 4.0.1). The mRNA expression of 8 markers were analyzed by GEPIA (version 2).

For manuscripts utilizing custom algorithms or software that are central to the research but not yet described in published literature, software must be made available to editors and reviewers. We strongly encourage code deposition in a community repository (e.g. GitHub). See the Nature Research guidelines for submitting code & software for further information.

Data

Policy information about availability of data

All manuscripts must include a data availability statement. This statement should provide the following information, where applicable:

- Accession codes, unique identifiers, or web links for publicly available datasets
- A list of figures that have associated raw data
- A description of any restrictions on data availability

The data that support the findings of this study are available within the paper and its Supplementary Information. Source data are provided with this paper. Expression data of mRNA transcripts of 8 protein markers in the TCGA TARGET GTEx cohort were accessed by publicly available database Xena (https://xenabrowser.net/datapages/?cohort=TCGA%20TARGET%20GTEx&removeHub=https%3A%2F%2Fxena.treehouse.gi.ucsc.edu%3A443).

Field-specific reporting

Please select the one below that is the best fit for your research. If you are not sure, read the appropriate sections before making your selection.

[x] Life sciences [ ] Behavioural & social sciences [ ] Ecological, evolutionary & environmental sciences

For a reference copy of the document with all sections, see nature.com/documents/nr-reporting-summary-flat.pdf

Life sciences study design

All studies must disclose on these points even when the disclosure is negative.

Sample size	For MBC detection, n = 123 clinical samples were used to differentiate MBC from NMBC and HD. For MBC monitoring, n = 147 samples were used to differentiate PD from PR/SD. The sample sizes were determined by sample availability. These sample sizes were sufficiently large for performing LDA classification based on previous experience that sample size is not less than 10 times the features (i.e., the expression level of 8 EV markers). These sample sizes also gave us enough statistical power as evidenced by narrow confidential intervals of accuracy (deviation from the central value was less than 15 %) and AUC (deviation from the central value was less than 0.15) for BC versus HD discrimination, MBC versus NMBC discrimination and PD versus PR/SD discrimination achieved by most individual markers and EV signature.
Data exclusions	No data were excluded.
Replication	TAS measurements of 3 protein markers in three types of samples and the evaluation of TAS performance were successfully reproduced using three independent replicates (Fig. 1d,e). In addition, the successful replication for TAS measurement in a clinical sample was shown by low inter-batch and intra-batch variations of less than 17 % (see Supplementary Fig. 11). Having proved the reproducibility, TAS measurement of the remained clinical samples was performed without replication.
Randomization	For treatment response monitoring, samples were randomly assigned to the training or validation cohort. For diagnosis, all samples were not divided and thus randomization was not involved. For prognosis, samples were not divided into the training or validation cohort and samples in prospective cohort were collected depending on sample availability, and thus randomization was not involved.
Blinding	The investigators who performed TAS assay and ELISA assay was blinded to cell line type, patient selection, and the diagnostic results and treatment responses of all cohorts.

Reporting for specific materials, systems and methods

We require information from authors about some types of materials, experimental systems and methods used in many studies. Here, indicate whether each material, system or method listed is relevant to your study. If you are not sure if a list item applies to your research, read the appropriate section before selecting a response.

Materials & experimental systems

n/a	Involved in the study	
[ ]	[x]	Antibodies
[ ]	[x]	Eukaryotic cell lines
[x]	[ ]	Palaeontology and archaeology
[x]	[ ]	Animals and other organisms
[ ]	[x]	Human research participants
[x]	[ ]	Clinical data
[x]	[ ]	Dual use research of concern

Methods

n/a	Involved in the study	
[x]	[ ]	ChIP-seq
[x]	[ ]	Flow cytometry
[x]	[ ]	MRI-based neuroimaging

Antibodies

Antibodies used	anti-CA15-3 antibody (Supplier name: CanAg, Catalog number: #200-10)<br>anti-CA125 antibody (Supplier name: CanAg, Catalog number: #400-10)<br>anti-CEA antibody (Supplier name: CanAg, Catalog number: # 401-10)<br>anti-CD63 antibody (Supplier name: CUSABIO, Catalog number: #CSB-E14107h)<br>anti-CD41 antibody (Supplier name: CUSABIO, Catalog number: #CSB-EL011865HU)
Validation	All antibodies used are commercially available and were validated either by manufacture or used in published research papers. For example:<br>Preservation of small extracellular vesicles for functional analysis and therapeutic applications: a comparative evaluation of storage conditions, Drug Delivery, 2021, 28:1, 162-170.<br>Disposable Reagentless Electrochemical Immunosensor Array Based on a Biopolymer/Sol-Gel Membrane for Simultaneous Measurement of Several Tumor Markers, Clinical Chemistry, 2008, 54:9, 1481–1488.

Eukaryotic cell lines

Policy information about cell lines

Cell line source(s)	Human breast cancer cell lines (BT-474, SK-BR-3, and MDA-MB-231) and human mammary epithelial cell line MCF-10A were obtained from ATCC.
Authentication	Cell types were authenticated via STR profiling and matched their publicly available STR profiles. All cell lines were used without any modification.
Mycoplasma contamination	All cell lines were negative for mycoplasma contamination.
Commonly misidentified lines (See ICLAC register)	No commonly misidentified cell lines were used.

Human research participants

Policy information about studies involving human research participants

Population characteristics	All the participants were female. For MBC diagnostics, 123 participants were older than 27 years and younger than 83 years, including 57 patients that had been diagnosed as BC with distant metastasis (MBC, n = 36) or without distant metastasis (NMBC, n = 21) and 66 age-matched healthy donors. Prior to sample collection, the MBC patients received no salvage treatment and the NMBC received no neoadjuvant treatment or surgery. Relevant information on the human participants in the MBC detection cohort are presented in Supplementary Table 4. For treatment monitoring, 147 samples were from MBC patients that were older than 28 years and younger than 80 years after 1 to 4 periods of treatment. Relevant information on the human participants in the MBC monitoring cohort are presented in Supplementary Tables 10 and 12. For PFS prediction, 75 MBC patients were older than 28 years and younger than 80 years. Relevant information on the human participants in the MBC prognosis cohort are presented in Supplementary Tables 14 and 15.
Recruitment	BC patients were recruited from the Fifth Medical Centre, Chinese PLA General Hospital. Healthy donors were recruited from the Second Medical Center, Chinese PLA General Hospital and the Fifth Medical Centre, Chinese PLA General Hospital. Only BC patients with definite information of gender, age, and pathological diagnosis were recruited. The study complied with all relevant ethical regulations and was approved by the Ethics Committee of the Fifth Medical Center of PLA General Hospital and Chinese PLA General Hospital Ethics Committee. All individuals were anonymized, and only gender, age, pathological diagnosis, treatment plan and treatment response were recorded. No self-selection criteria bias for patient populations was anticipated.
Ethics oversight	The study is conducted in accordance with Declaration of Helsinki protocol and in accordance with the terms and conditions of the ethical approval from the Ethics Committee of the Fifth Medical Center of PLA General Hospital and Chinese PLA General Hospital Ethics Committee.

Note that full information on the approval of the study protocol must also be provided in the manuscript.
